# Reduced Hippocampal Functional Connectivity During Episodic Memory Retrieval in Autism

**DOI:** 10.1093/cercor/bhw417

**Published:** 2017-01-05

**Authors:** Rose A. Cooper, Franziska R. Richter, Paul M. Bays, Kate C. Plaisted-Grant, Simon Baron-Cohen, Jon S. Simons

**Affiliations:** 1 Department of Psychology, University of Cambridge, Cambridge, CB2 3EB, UK; 2 Autism Research Centre, Department of Psychiatry, University of Cambridge, Cambridge, CB2 8AH, UK

**Keywords:** autism, connectivity, fronto-parietal control network, hippocampus, memory retrieval

## Abstract

Increasing recent research has sought to understand the recollection impairments experienced by individuals with autism spectrum disorder (ASD). Here, we tested whether these memory deficits reflect a reduction in the probability of retrieval success or in the precision of memory representations. We also used functional magnetic resonance imaging (fMRI) to study the neural mechanisms underlying memory encoding and retrieval in ASD, focusing particularly on the functional connectivity of core episodic memory networks. Adults with ASD and typical control participants completed a memory task that involved studying visual displays and subsequently using a continuous dial to recreate their appearance. The ASD group exhibited reduced retrieval success, but there was no evidence of a difference in retrieval precision. fMRI data revealed similar patterns of brain activity and functional connectivity during memory encoding in the 2 groups, though encoding-related lateral frontal activity predicted subsequent retrieval success only in the control group. During memory retrieval, the ASD group exhibited attenuated lateral frontal activity and substantially reduced hippocampal connectivity, particularly between hippocampus and regions of the fronto-parietal control network. These findings demonstrate notable differences in brain function during episodic memory retrieval in ASD and highlight the importance of functional connectivity to understanding recollection-related retrieval deficits in this population.

## Introduction

Autism spectrum disorder (ASD) is most notably characterized by difficulties with social interaction and communication as well as restrictive and repetitive behaviors, but is also associated with a specific profile of episodic memory deficits, involving explicit retrieval of previously experienced events (Tulving 1985). In particular, several strands of evidence demonstrate that high-functioning ASD is associated with subtle but relatively selective deficits in the process of recollection but preserved familiarity-based memory (see Boucher et al. 2012 and Bowler et al. 2011 for discussions). Recollection-based recognition memory involves memory for specific details or the context in which a studied item is experienced, whereas familiarity involves knowing an item has been encountered before but without recollection of additional details of its original context (Yonelinas, 2002). Moreover, there is evidence for both encoding and retrieval-related influences on recollection deficits in ASD. For instance, some studies have suggested that individuals with ASD have difficulty in organizing information to be learnt (Bowler et al. 2008; Gaigg et al. 2008) and initiating strategic encoding processes (Meyer et al. 2014). On the other hand, memory deficits in ASD are observed during explicit but not implicit memory retrieval (Ring et al. 2015) and episodic memory difficulties are substantially attenuated when cognitive control and retrieval demands are minimized (Bowler et al. 2004; Crane et al. 2013; Maras et al. 2013; Solomon et al. 2016), thus implying a distinct difficulty in engaging the explicit process of recollection during retrieval. Despite some progress in characterizing the cognitive mechanisms influencing recollection in ASD, the specific characteristics of this recollection deficit, namely an impairment in the probability of recollection and/or the fidelity of recollected information, and its neural underpinnings are however currently unknown.

The majority of episodic memory studies in ASD to date have focused on binary distinctions of recollection success or failure, such as “remembering” or “knowing” that an item was previously encountered (Bowler et al. 2007; Meyer et al. 2014; Cooper et al. 2015), retrieving the source context in which an item was studied (Bowler et al. 2004; Cooper et al. 2016), or identifying similar items as old or new (Cooper et al. 2015; Ring et al. 2016). However, a novel strand of research in typical individuals has used continuous measures of retrieval to demonstrate a wide range in the fidelity of successfully remembered memory representations, suggesting that retrieval precision may be a separable aspect of short- and long-term memory that varies in a graded fashion (e.g. Bays et al. 2009; Brady et al. 2013; Harlow and Donaldson 2013; Harlow and Yonelinas 2014; Richter et al. 2016). Research in ASD has yet to examine retrieval precision directly, but there are hints of a reduction in memory quality in terms of reduced specificity of autobiographical event details (Crane et al. 2012), reduced memory salience (Lind et al. 2014), and reduced confidence in correct memories (Grainger et al. 2014). Furthermore, individuals with ASD show a reduction in the number of eye movements to previously studied regions of scenes during successful recollection (Cooper et al. 2017), suggesting less reconstruction of event details. Findings of impaired source memory and a reduced ability to discriminate similar items could be partially driven by impoverished precision of memories, leading 2 sources or items to appear more similar in memory. The use of continuous retrieval measures to dissociate memory success and precision could thus provide a novel approach to pinpoint the nature of episodic memory retrieval deficits in ASD.

Another key method for furthering our understanding of the neurocognitive basis of episodic memory deficits in ASD is functional magnetic resonance imaging (fMRI). Within typical individuals, a broad network of regions is thought to play a role in episodic memory retrieval, including the medial temporal lobe, posterior parietal cortex (PPC), and prefrontal cortex (e.g. Spaniol et al. 2009; Rugg and Vilberg 2013; Kim 2016). The prefrontal cortex has been associated with strategic encoding and retrieval, working memory, and postretrieval monitoring of memory representations (e.g. Fletcher and Henson 2001; Badre and Wagner 2007; Blumenfeld and Ranganath 2007), the hippocampus (HC) has been associated with relational encoding (Konkel and Cohen 2009) and recollection (Eichenbaum et al. 2007), and PPC is thought to represent memory details online during retrieval (Vilberg and Rugg 2012; Kuhl and Chun 2014; Bonnici et al. 2016). In a recent study involving neurotypical individuals, we identified a dissociation between the HC and PPC in terms of their roles in memory success and memory precision, respectively (Richter et al. 2016). These findings are especially relevant given that behavioral research has indirectly implicated all of the aforementioned brain regions in episodic memory impairments in ASD, including a hippocampal relational encoding deficit (Bowler et al. 2014), reduced subjective memory quality as a result of parietal dysfunction (cf. Boucher and Mayes 2012), and increased memory deficits with task complexity (Bowler et al. 2004; Solomon et al. 2016) and reduced ability to integrate and monitor information in memory (Ben Shalom 2009; Cooper et al. 2016) due to prefrontal dysfunction. The authors of behavioral studies have debated the neural basis of memory deficits in ASD (cf. Bowler et al. 2010; Solomon et al. 2011; Cooper et al. 2015), but these theories remain largely untested at a neural level.

In fact, only 2 studies to date have used fMRI to investigate brain activity differences during episodic memory in ASD, and both addressed encoding rather than retrieval. Each study observed atypical encoding-related lateral prefrontal activity in ASD, with one study providing evidence for overall attenuated frontal activity and a negative relationship between frontal activity and subsequent memory (Greimel et al. 2012) and the other finding enhanced lateral frontal activity but no relationship between frontal activity and subsequent recollection in ASD in contrast to the positive relationship observed in typical individuals (Gaigg et al. 2015). Thus, current findings are rather inconsistent and provide limited insight into the neural processes associated with recollection dysfunction during retrieval in ASD. Other insights that may be relevant to understanding the neural basis of episodic memory in ASD are provided by a number of fMRI studies of executive function and working memory, which observed reduced frontal and parietal activity during learning, working memory, and problem solving in ASD (Koshino et al. 2008; Soulieres et al. 2009; Damarla et al. 2010; Solomon et al. 2015), and that individuals with ASD exhibited less of an increase in activity of these regions with increasing task complexity compared with typical individuals (Solomon et al. 2009; Yamada et al. 2012; Vogan et al. 2014; Simard et al. 2015). Therefore, it is possible that dysfunction of frontal and parietal regions may contribute to reduced top-down control of recollection-based memory retrieval in ASD.

Going beyond regional effects, recent imaging research in ASD indicates that information-processing differences, including in episodic memory, are best explained by differences in functional connectivity rather than in region-specific activity (Just et al. 2012; Barendse et al. 2013). A number of studies in ASD have demonstrated reduced task-related functional connectivity within the fronto-parietal task control network (FPCN) (e.g. Solomon et al. 2009; Damarla et al. 2010; Yamada et al. 2012), which includes lateral prefrontal and inferior parietal cortices. For example, individuals with ASD have been found to exhibit similar regional brain activity to typical individuals, but reduced functional connectivity between frontal and posterior brain regions, during implicit learning (Schipul and Just 2016). Similarly, reduced intrinsic default mode network (DMN) connectivity, including regions such as medial prefrontal, posterior cingulate, and posterior parietal cortices, has been related to working memory deficits in ASD (Chien et al. 2016). Moreover, episodic memory research within the typical population has increasingly focused on brain network dynamics as an important moderator of episodic memory retrieval. Functional connectivity strength of whole-brain networks, particularly involving important “hubs” such as the HC and medial prefrontal cortex (MPFC, part of the DMN), seems to be particularly important for episodic memory retrieval (Huijbers et al. 2011; Robin et al. 2015) and for promoting retrieval success (Schedlbauer et al. 2014; King et al. 2015; see Preston and Eichenbaum 2013 for a review). FPCN connectivity is additionally increased during conscious recollection (Schedlbauer et al. 2014; see Simons and Spiers 2003 for a review) and dynamic increases in connectivity between DMN and FPCN facilitate flexible goal-directed behavior (Spreng et al. 2010; Zanto and Gazzaley 2013) that contributes to episodic memory retrieval (Fornito et al. 2012; Westphal et al. 2016). Widespread increases in functional connectivity, particularly involving hubs such as the HC, therefore seem to be important for facilitating episodic memory retrieval. No study to date, however, has investigated functional connectivity during long-term memory encoding and retrieval in ASD.

In the present study, adults with ASD and typical controls completed a memory task in which they used a continuous dial to recreate the features of previously encoded visual displays. Behavioral and retrieval-related fMRI data from the neurotypical control participants in the current study were presented in our recent paper (Richter et al. 2016). In the present analysis, fMRI scanning allowed us to test for differences between individuals with ASD and typical controls in both memory encoding- and retrieval-related neural activity, as well as effects specific to retrieval success and precision. Additionally, functional connectivity of a priori defined episodic memory regions and 2 important brain networks, the FPCN and DMN, was assessed during both the memory encoding and retrieval tasks. It was predicted that the ASD group would show a reduction in retrieval success, in line with previous findings of reduced instances of recollection (e.g. Bowler et al. 2007; Cooper et al. 2015) but that individuals with ASD would also show impoverished retrieval precision for successfully retrieved memories. Based on previous fMRI evidence, we expected a reduced relationship between lateral prefrontal encoding activity and subsequent memory in the ASD group (cf. Gaigg et al. 2015). During memory retrieval, frontal and possibly parietal activity was expected to be reduced in ASD, reflecting a difficulty in accessing and monitoring memory representations during retrieval. We did not expect any group differences in medial temporal activity (cf. Cooper et al. 2015; Solomon et al. 2015, 2016). Moreover, reduced connectivity was predicted between core regions of the memory network and whole-brain networks, including the FPCN and DMN, during memory retrieval in line with the theory that underconnectivity substantially contributes to information-processing deficits in ASD (Just et al. 2012).

## Materials and Methods

### Participants

Forty-eight participants aged between 18 and 45 took part in the current study, including 24 adults with ASD (14 males) and 24 healthy control participants (13 males). Participants had normal or corrected-to-normal vision and hearing, were not color blind, and spoke fluent English. None of the control participants had diagnoses of any psychiatric, neurological, or developmental disorder or learning difficulty, and participants in the ASD group had a formal diagnosis of autism according to DSM-5 or ICD-10 criteria, having received their diagnosis following specialist assessment by a qualified clinician. Additional criteria for fMRI scanning included being right-handed and having no metal implants or other medical issues that would prevent participants from being scanned. Twenty (11 males) out of the total 24 ASD participants met the inclusion criteria to be scanned and so 20 (11 males) out of the 24 control participants were also scanned to match the groups in terms of sample size.

All participants were administered the Autism Spectrum Quotient (AQ; Baron-Cohen et al. 2001), as well as Raven's Advanced Progressive Matrices (short-form) (Arthur and Day 1994) and the National Adult Reading Test (NART) (Nelson 1982) as indices of nonverbal and verbal ability, respectively. Control participants were group-matched to ASD participants on age, education, verbal and nonverbal ability (see Table 1). The subsamples of ASD and control participants who were scanned were also matched on all the aforementioned variables. For 2 scanned participants (1 control, 1 ASD), 1 block out of the 8 task blocks was lost due to scanner error and so only 7 out of 8 task blocks were analyzed. In addition to a final sample of 20 participants per group for the fMRI analyses and 24 participants per group for the behavioral analyses, 1 additional control participant was scanned but was excluded due to consistent chance-level performance, and 2 additional ASD participants did not complete the scanning session due to persistent movement during the scan and pressing the alarm buzzer due to discomfort, respectively. Importantly, participants in the ASD and control groups who were scanned did not differ in the amount of head movement throughout the task, in terms of the mean distance moved (absolute change in mm) in any direction or the mean rotation (absolute change in degree) (*t*s(38) < 0.6, *P*s > 0.5). There was also no evidence for between-group differences in movement variability (standard deviation, SD) (*t*s(38) < 0.5, *P*s > 0.6) or maximum movement (*t*s(38) < 1, *P*s > 0.3).

**Table 1:** Demographic details for the control and ASD groups: mean (SD)

	Control behavioral (*N* = 24)	ASD behavioral (*N* = 24)	*P*	Control scanned (*N* = 20)	ASD scanned (*N* = 20)	*P*
Age	29.6 (6.2)	30.3 (8.4)	0.77	29.8 (6.7)	30.9 (8.9)	0.66
Education	16.9 (1.8)	16.6 (1.9)	0.54	16.7 (1.9)	16.4 (1.8)	0.55
NART	35.1 (6.4)	33.3 (5.7)	0.29	35.8 (6.6)	33.3 (5.9)	0.21
Raven's	10.8 (1.1)	10.5 (1.5)	0.45	10.8 (1.1)	10.4 (1.6)	0.26
AQ	16.6 (6.1)	37.6 (7.0)	<0.001	15.4 (5.8)	36.4 (6.9)	<0.001

Participants with ASD were recruited via participant databases at the Cambridge Laboratory for Research into Autism and the Autism Research Centre, Cambridge. Control participants were recruited via participant databases at the Behavioural and Clinical Neuroscience Institute (BCNI) and Memory Laboratory, Cambridge University, as well as social media adverts. Ethical approval was obtained from the Cambridge Psychology Research Ethics Committee. Participants gave written informed consent prior to taking part and were paid a standard honorarium for their time. An analysis of data from the 20 control participants who underwent fMRI scanning has been published in our recent paper (Richter et al. 2016).

### Procedure

**Figure 1.**
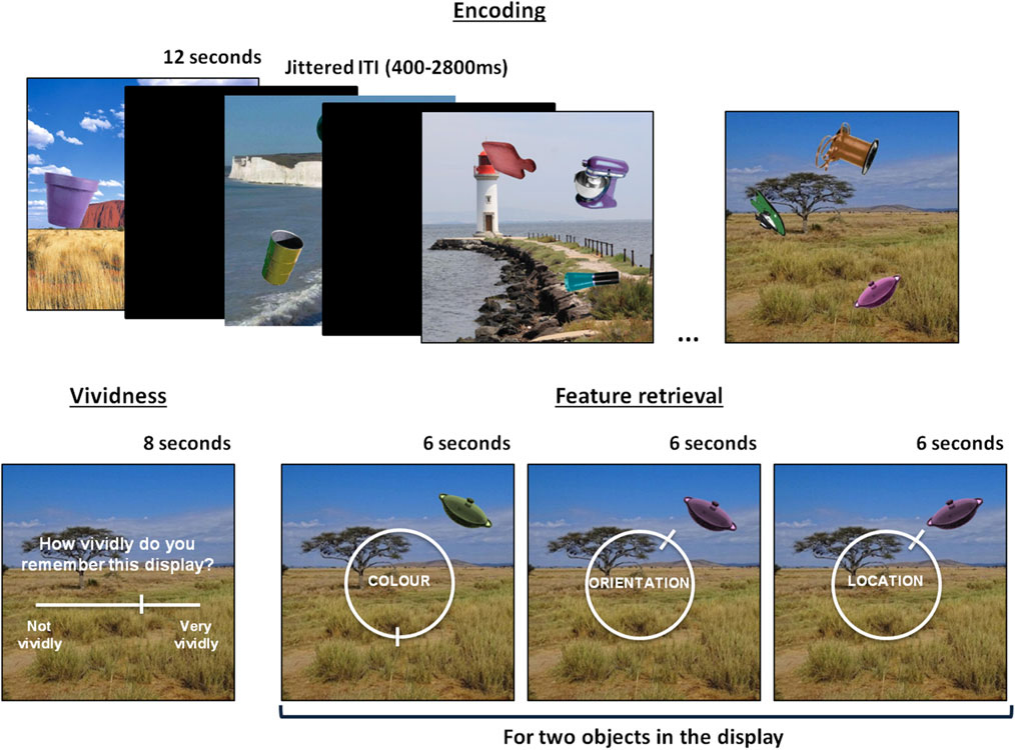
An illustration of the task design, showing a series of displays learnt during encoding and the subsequent memory test on an individual display during the retrieval phase.

fMRI scanning was completed at the MRC Cognition and Brain Science Unit, Cambridge, and behavioral testing was conducted at the BCNI, Cambridge. The memory task was programmed using Psychtoolbox and participants who were not scanned listened to scanner noise through headphones while completing the task to keep noise-related attentional distraction comparable. The memory task was divided into 8 encoding-retrieval blocks and both phases of the blocks were scanned. Participants studied a total of 48 displays (750 × 750 pixels), each containing a background scene overlaid with 3 everyday objects (obtained from http://timbrady.org/stimuli/ColorRotationStimuli.zip, Brady et al. 2013) varying on 3 features: color, orientation, and location (see Fig. 1). Colors, orientations, and locations of all objects were selected pseudo-randomly from circular parameter spaces, with the constraint that at least 62° would separate the same feature of different objects on the display, which was enforced so that locations did not overlap. All object-background assignments were randomized and displays were kept consistent across subjects. Presentation order of the displays during the study phase was randomized per participant and displays were studied for 12 s each. Participants were told to try to learn the appearance of the objects on the background as best as they could in preparation for a memory test on the object features. A 10-s delay followed each encoding phase before the retrieval phase started.

During the memory retrieval phase (see Fig. 1), participants were first presented with the background scene of a studied display and were asked to rate how vividly they remembered the appearance of the objects associated with the presented background, using a continuous slider from “not vividly” (0) to “very vividly” (100) that appeared 2 s after the question onset. Participants were then tested on their memory for 2 of the 3 objects that were associated with a given display. Each tested object was initially presented in a random color, orientation, and location. The participants’ task was to sequentially recreate the objects’ original features (color/orientation/location) as precisely as they could remember them over 6 trials (3 features for 2 objects). The order in which the features were tested was fully counterbalanced. Responses were given by moving around a continuous 360° response space represented by a circular dial. Specifically, participants were instructed to hold down response buttons under their index and middle fingers to move the pointer left and right, respectively, around the circular dial on the screen. Subjects had up to 6 s to respond to each memory retrieval question by pressing a button under their thumb to confirm their response. All trials were separated by a fixation cross of jittered duration (400–2800 ms, mean = 961 ms).

### Behavioral Analyses

To derive separate estimates of retrieval success and retrieval precision, a mixture model was fitted to the aggregate error data (target feature—response) across all 6876 trials from participants within each group, including a von Mises component, which is a circular Gaussian distribution capturing “correct” responses centered on the target (encoded) feature value (Zhang and Luck 2008; Bays et al. 2009), as well as a uniform component, capturing random responses evenly distributed from 0° to 360° (consistent with previous studies of precision in long-term memory, e.g. Harlow and Donaldson 2013; Richter et al. 2016). Of note, this mixture model was demonstrated to fit the current long-term memory data better than other models in our recent paper (Richter et al. 2016). Retrieval success was operationalized as the proportion of responses that fit the von Mises distribution versus the uniform distribution, and precision as the concentration of the von Mises distribution. The aggregate data from each group were modeled (as has been recently done in another study of long-term retrieval precision, Harlow and Yonelinas 2014), rather than individual subjects’ data, because of instances of poor model fit and unreliable parameter estimates for individual participants with variable performance. Analyzing the aggregate data had the additional advantage of making it possible to generate performance estimates for the 3 features (color/orientation/location), which would have been difficult on the single subject level due to lower trial numbers. Permutation tests were used to statistically compare the aggregate group data, wherein participants were randomly shuffled and regrouped over 1000 iterations and the mixture model was fitted to the groups’ data from each iteration. A distribution of group differences was calculated from the 1000 iterations and the *P* value (2-tailed) was calculated as the proportion of iterations where the absolute group difference exceeded the difference between the control and ASD groups.

### fMRI Analyses

#### Data Acquisition and Preprocessing

fMRI scanning was performed using a 3-T Siemens TIM Trio scanner and a 32-channel head coil. Structural images were obtained using a *T*_1_-weighted protocol (256 × 256 matrix, 192 1 mm sagittal slices, TR = 2.25 s, TE = 3 ms) and functional images were acquired approximately parallel to the Anterior Commissure–Posterior Commissure transverse plane using a single-shot Echo Planar Imaging (EPI) sequence (TR = 2 s; TE = 30 ms; field of view = 192 × 192 mm, flip angle = 78°). Functional scans were obtained as 32 contiguous oblique-axial slices (3 × 3 × 3 mm voxels) per volume. Each of the 8 fMRI scan runs (encoding-retrieval block) contained a total of 205 volumes, and the first 5 volumes were discarded from each scan run to allow for stabilization of the magnetic field.

Data preprocessing and analysis was performed using SPM12 (Wellcome Department of Imaging Neuroscience, University College London, London, UK), implemented via an automatic analysis pipeline (Cusack et al. 2015) (version 4; http://www.automaticanalysis.org) as well as custom Matlab scripts. For each participant, the structural image was coregistered to the SPM Montreal Neurological Institute (MNI) template and then bias corrected to control for intensity differences due to inhomegeneities. The structural image was then segmented into different tissue classes (gray matter, white matter, and cerebrospinal fluid) using SPM's unified segmentation approach. The individual subject's tissue class images from this segmentation step were used to create a custom template structural image using DARTEL (Diffeomorphic Anatomical Registration Through Exponentiated Lie Algebra). The structural images were transformed to MNI space, and finally, were smoothed with an 8 mm full-width at half-maximum (FWHM) Gaussian kernel. The functional images were motion-corrected and were realigned to the middle functional image to correct for effects of slice timing. The EPI images were then coregistered to the structural image and normalized to MNI space using the DARTEL template. Functional images were smoothed using an 8 mm FWHM Gaussian kernel.

#### Regions of Interest

Analyses of blood oxygen level-dependent (BOLD) activity and functional connectivity were focused on 4 a priori defined regions of interests (ROIs) that play a role in episodic memory (e.g. Spaniol et al. 2009; Rugg and Vilberg 2013; Kim 2016; Westphal et al. 2016) and have been commonly discussed in relation to episodic memory deficits in ASD (e.g. Bowler et al. 2011; Boucher et al. 2012; Solomon et al. 2016): MPFC, lateral prefrontal cortex (LPFC), HC, and PPC. ROIs were created using peak coordinates from a recent meta-analysis of episodic memory retrieval (Kim 2016) within the left-hemisphere. Of note, 14 mm spheres were generated around each of the peak coordinates to form the ROIs, with this size chosen due to evidence from functional connectivity that voxels within 14 mm form a “local” network and are highly correlated with one another (Sepulcre et al. 2010). ROIs were additionally masked with broad anatomical masks of regions within the medial temporal lobe (HC and parahippocampal gyrus), lateral parietal cortex, and frontal cortex generated using the Wake Forest University (WFU) pick atlas, selected from the automated anatomical labeling atlas, to ensure all voxels fell within these broad regions. Small-volume correction at a voxel-wise height threshold of *P* < 0.05 family-wise error corrected was applied within each ROI when assessing the statistical significance of BOLD activity ROI results.

#### Univariate General Linear Model

A general linear model (GLM) was constructed containing separate regressors corresponding to the 6 different event types: encoding trials, vividness trials, 3 separate regressors for successfully retrieved features (color/orientation/location), as well as one regressor for any unsuccessfully retrieved features. Unsuccessful trials from all features were modeled together due to low trial numbers for some participants. At the first level, trials were modeled for each participant by convolving a boxcar function (corresponding to the event durations from the onset of each event) with the canonical haemodynamic response function. The durations used to model the data were 12 s for the encoding displays, 8 s for the vividness rating, and 6 s for the feature questions. In order to estimate neural activity associated with successful memory retrieval, it was necessary to determine whether each individual trial was likely to have been successfully remembered (some information recalled, regardless of how precise) or forgotten (no feature information recalled). To do so, we modeled the aggregate data across all participants and both groups and, using the probability density functions of the von Mises and uniform distributions, estimated the point at which the slope of the von Mises distribution approached zero by determining where the probability of a response fitting the von Mises distribution reached 5% (resulting in a cut-off of ±62° from the target value). The same threshold was used across all features and for all participants so that differences in neural correlates of success and precision were not biased by the range of data available for analysis.

Parametric modulators were additionally included to model effects of subsequent memory success, vividness and precision, and all parametric modulators were mean-centered (so that the average value of each regressor was zero) prior to modeling. Parametric modulators were included for all regressors with the exception of unsuccessfully retrieved trials, for which any variation in retrieval precision was expected to be random as a result of guessing and should thus not be meaningfully correlated with neural activity. The parametric modulator for encoding trials reflected the number of subsequently retrieved features (0–6), trial-by-trial vividness ratings were included as a parametric modulator for vividness trials, and lastly the precision of the response was included as a parametric modulator for successfully retrieved trials. Trial-specific estimates of retrieval precision were obtained by subtracting the error (absolute distance between the target value and the response) from 180 (the maximum possible error), so that higher values reflected higher precision. Time and dispersion derivatives were included for each event and each parametric modulator. For the GLM, the 8 separate scan blocks were concatenated for analysis to obtain more stable parameter estimates from increasing the number of trials per condition (e.g. Uncapher et al. 2006; Bergström et al. 2015). A high pass filter was applied by including 6 additional regressors for each block representing a Discrete Cosine Transform (DCT) set capturing frequencies up to 1/128 Hz. Six regressors representing motion were included per block and 8 constant regressors were included to model differences between the blocks.

Subject-specific effects from each first-level contrast were then entered into second-level, random effects analyses, with each group entered as a separate regressor. For all ROI analyses of BOLD activity, main effects of event type (effects contrasted with the implicit baseline) and event type x group interactions were analyzed for each contrast of interest. Contrasts were focused on identifying both task-based and performance-related differences in BOLD activity. First, activity during the encoding task (vs. implicit baseline), regardless of subsequent memory, was compared between the groups, and then, using the subsequent memory parametric modulator, regions where activity positively predicted subsequent retrieval success were compared between groups. To estimate task differences during retrieval, regional BOLD activity during the retrieval of the object features (vs. implicit baseline) was compared between groups, then the groups were compared on neural changes positively associated with successful (vs. unsuccessful) retrieval as well as the precision of successfully recalled features. All analyses of memory retrieval were conducted across the 3 features (color/orientation/location) so that fMRI effects reflected the underlying memory process tested, rather than memory for a particular feature.

#### Functional Connectivity Analyses

Functional connectivity analyses were conducted using the beta-series correlation method (Rissman et al. 2004). At the first level, each participant's data was modeled using a GLM wherein each trial was modeled as an individual regressor. This resulted in a model with 384 trial-specific regressors for each participant. DCT, motion, and block constant regressors were included as for the original univariate analyses described above. Every voxel, therefore, had a resulting beta series of 384 beta values, one per trial, which were divided into task-specific beta series (e.g. encoding task, feature retrieval task). The degree to which 2 regions in the brain interact during a task is quantified by Pearson's correlation between their beta series. The aforementioned ROIs were used to define seed regions for connectivity analyses. Seed regions were defined on a participant-specific basis as 5 mm spheres centered on each participant's peak BOLD activity within each ROI from the initial univariate GLM for a given contrast of interest (see Results for the contrasts used). Each seed's beta series was calculated as the mean beta series of all voxels within the 5 mm sphere.

To characterize connectivity in the current study, we first focused on the overall level of connectivity between each seed region and the rest of the brain by calculating a measure of whole-brain connectivity strength for each task. Recent evidence suggests that a summary measure of whole-brain connectivity strength is valuable for considering the general importance of regions (or hubs) and the efficiency of their communication within a whole-brain network during memory performance (Schedlbauer et al. 2014; Geib et al. 2015), especially given that widespread increases in connectivity between core memory regions and many regions across the whole brain appear to be important for episodic memory retrieval (Geib et al. 2015; King et al. 2015). This analysis, therefore, allowed us to establish the degree to which our seed regions were important within a whole-brain network during the memory task in our ASD and control groups. To derive a measure of whole-brain connectivity strength, the brain parcellation method developed by Power et al. (2011) was used that, based on resting state data, characterized 264 functional nodes across the whole brain. Of note, 5 mm spheres were generated around all of the coordinates identified by Power et al., consistent with the original study and another recent investigation of memory retrieval using this parcellation method to investigate connectivity between ROIs and whole-brain networks (Westphal et al. 2016). Across all participants, 39 nodes frequently contained fewer than 5 valid voxels and so were removed from analyses. This resulted in a total of 225 nodes used across all subjects to characterize whole-brain connectivity. To obtain a measure whole-brain connectivity strength for each seed region, an adapted measure of weighted degree centrality (node strength) from graph theory was used (e.g. Bullmore and Sporns 2009; Rubinov and Sporns 2010; Zuo et al. 2012). This measure represents the proportion of “connections” between a region and all other nodes within the brain, weighted by strength of those connections:

$$C_{i}=\frac{\sum_{j\in A}^{N}r_{\mathrm{ij}}}{N}$$

where the connectivity strength (*C*) for seed *i* is the sum of all node connections (*r*_*ij*_) that are above the connection threshold (indicated by *A*) divided by the total number of nodes (*N*) (possible connections). Meaningful connections are defined as any correlation greater than *r* = 0.25 to remove the influence of signal noise creating artificial above-zero correlations. This threshold approach was based on that implemented by Buckner et al. (2009) and is commonly applied when calculating degree centrality measures. Beta-series functional connectivity analysis has also previously been used successfully in conjunction with graph theory measures (Schedlbauer et al. 2014; Geib et al. 2015).

Having identified any potential differences in whole-brain connectivity strength of our seed regions, we then aimed to further specify differences in connectivity by investigating whether such seed connectivity differences could be most attributed to connectivity with either or both of 2 large-scale networks commonly discussed in relation to memory: the DMN and FPCN. Out of the 225 nodes across the brain, Power et al. (2011) identified 55 as belonging to the DMN and 24 as belonging to the FPCN. Due to recent evidence that the specific regions involved in large-scale networks do not fully overlap between resting state cognitive tasks (cf. Bellana et al. 2016), a further step was taken to ensure that the DMN and FPCN networks represented connected regions during our memory task by checking that, for each network node, within-network connectivity was higher than between-network connectivity. Node strength from the seed regions to each network was calculated using the aforementioned calculation but limited to nodes within an individual network. Lastly, a whole-brain seed-to-voxel connectivity analysis was conducted to further specify individual regions that exhibited significant differences in connectivity between the groups for individual tasks. A threshold of *P* < 0.001, minimum extent of 20 voxels, was used to assess significant differences in connectivity. All Pearson's *r* correlation coefficients were Fisher's *z* transformed prior to any averaging across subjects and statistical analyses.

## Results

### Behavioral Results

Prior to distinguishing between memory success and precision, the mean performance on the task was compared between groups by contrasting the mean error (absolute value of target—response), using a 2 (group) × 3 (feature) ANOVA (see Table 2). In line with our prediction, the ASD group performed worse than the control group in recall of object features, *F*_1,46_ = 4.08, *P* < 0.05, *η*² = 0.08. A difference between features, *F*_2,92_ = 136.36, *P* < 0.001, *η*² = 0.74, reflected the observation that location was easier than both other tasks (*t*s(47) > 10.2, *P*s < 0.001, *d*s > 1.4) and color was also easier than orientation (*t*(47) = 4.6, *P* < 0.001, *d* = 0.66), and these effects did not differ between groups, *F*_1,46_ < 1, *P* > 0.4, *η*² < 0.01. The groups also did not differ significantly on mean memory vividness rating (control: mean = 49.18, SD = 14.63; ASD: mean = 46.15, SD = 16.85; *t*(46) = 0.66, *P* = 0.51, *d* = 0.19) and data from the vividness trials will not be further explored here.

**Table 2:** Mean absolute error (SD) across participants within each group, and model-estimated values of retrieval success and precision derived from the model of aggregate data

	Control			ASD		
	Mean error	Success (*pT*)	Precision (*k*)	Mean error	Success (*pT*)	Precision (*k*)
All	41.2 (12.2)	0.63	10.42	49.1 (140.5)	0.53	9.67
Col	45.4 (16.1)	0.62	6.02	51.4 (18.2)	0.55	5.06
Ori	52.5 (14.5)	0.49	13.15	62.9 (16.0)	0.37	13.96
Loc	25.6 (11.5)	0.80	13.87	33.0 (15.0)	0.72	11.99

Note: Performance measures are provided across all features as well as separately for each feature (Col = color, Ori = orientation, Loc = location).

**Figure 2.**
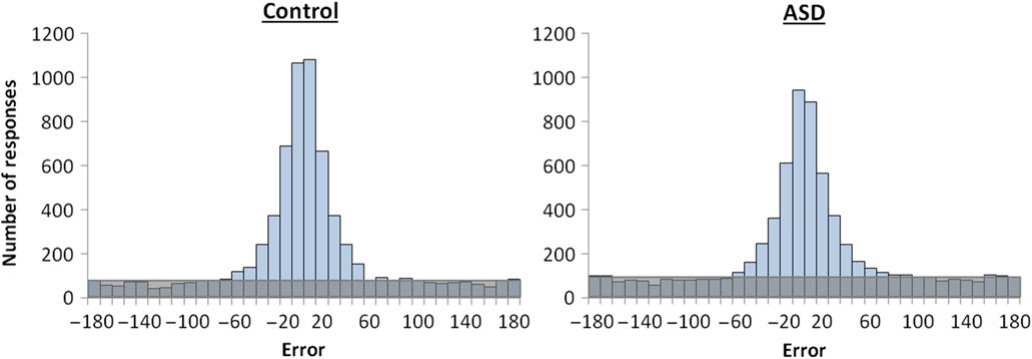
Error distributions across all participants (and all features) within the control and the ASD groups. The number of responses that are distributed uniformly from −180 to 180° (shaded area) is indicative of increased guessing (and lower retrieval success rate) in the ASD group, but the concentration parameter *k* (describing the response variability) of the von Mises component is similar across the 2 groups.

Given that higher mean error (worse performance) in the ASD group could be influenced by either impaired retrieval success, retrieval precision, or both, the next analysis sought to distinguish and compare estimates of retrieval success and retrieval precision between the groups. This was achieved by comparing the mean model-estimated values of proportion correct (proportion of target responses, *pT*) and precision (concentration parameter *k*, reflecting the width of the error distribution) across all retrieval trials (see Table 2). The aggregate error distributions for all 6876 trials in each of the groups can be seen in Figure 2. As 1 participant in each group provided data for only 7 out of 8 blocks, 6876 is the total number of retrieval trials for 23 participants with 8 blocks of data (each with 6 encoding displays × 6 feature questions) and 1 participant with 7 blocks of data per group. The ASD group showed a significant reduction in retrieval success compared with the control group (permutation *P* value = 0.04). Of note, while we favored the aggregate data analysis due to noisy single subject data, a parallel result was seen even when the modeling was performed for individual subjects (control: mean = 0.64, SD = 0.15; ASD: mean = 0.54, SD = 0.19; *t*(46) = 1.89, *P* = 0.06, *d* = 0.55). Conversely, we found no evidence for a reduction in retrieval precision (the fidelity of successfully remembered features) in the ASD group compared with the control group (permutation *P* value = 0.54), in line with the result when comparing the mean retrieval precision between groups when modeled individually (control: mean = 10.55, SD = 3.51; ASD: mean = 9.62, SD = 3.56; *t*(46) = 0.92, *P* = 0.36, *d* = 0.26). Group differences on aggregate retrieval success and retrieval precision values were converted to *z* scores to facilitate a direct comparison of the magnitude of group differences on the 2 retrieval measures; however, the reduction in retrieval success in ASD was not found to be disproportionately greater than the group difference in retrieval precision (permutation *P* value = 0.25). Both groups showed only a modest correlation between individual estimates of retrieval success and retrieval precision modeled across all features (control: *r* = 0.56; ASD: *r* = 0.40), with no difference in the magnitude of this relationship between groups (*z* = 0.68, *P* = 0.50), supporting the separability of retrieval success and precision in the ASD group as well as in the control group. Of note, the means for the subsample of scanned participants (20 ASD and 20 control) were very similar to the means obtained from the full sample for both success (control: mean = 0.62, SD = 0.16; ASD: mean = 0.53, SD = 0.20) and precision (control: mean = 10.05, SD = 3.41; ASD: mean = 9.43, SD = 3.40). It is also worth noting that the pattern described above remained relatively consistent across the 3 individual features when the aggregate data were modeled separately for color, orientation, and location (see Table 2).

### ROI BOLD Activity During Memory Encoding and Retrieval

**Figure 3.**
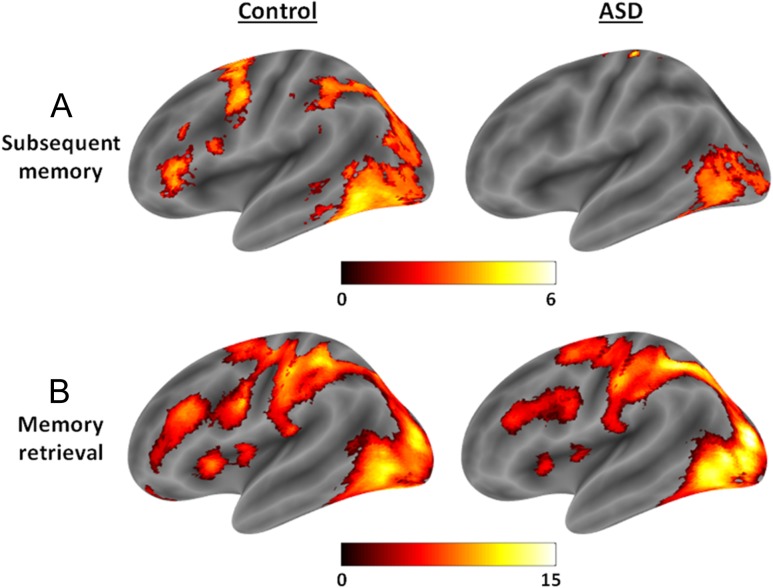
(*A*) Subsequent memory effects in the control and ASD groups, where the ASD group shows an attenuated relationship between LPFC activity and subsequent retrieval success (0–6 features recalled). (*B*) Activity during the memory retrieval task, where the ASD group shows attenuated LPFC activity relative to the control group. Effects are displayed using bspmviewer at a threshold of *P* < 0.01 uncorrected for visualization, with the scale reflecting *t* values.

To analyze potential group differences in BOLD activity during the memory task, the groups were first compared on ROI activity during the encoding task and subsequently on ROI activity during the memory retrieval task. During the memory encoding task, 3 ROIs showed a reliable increase in activity relative to baseline across participants of both groups, including LPFC (*t*(38) = 8.01, *P* < 0.001, peak: −45, 36, 6), HC (*t*(38) = 9.12, *P* < 0.001, peak: −30, −36, −15), and PPC (*t*(38) = 12.12, *P* < 0.001, peak: −24, −69, 39), but MPFC activity did not increase significantly (*t*(38) < 1.1, *P* > 0.7). There were no significant differences between the 2 groups in brain activity during the memory encoding task within any of the ROIs (*t*s(38) < 2.9, *P*s > 0.1). Subsequent memory effects were analyzed by measuring the correlation between trial-by-trial activity during encoding and subsequent retrieval success. Significant correlations between activity and subsequent memory were found in LPFC (*t*(38) = 4.52, *P* = 0.005, peak: −48, 36, 9) and PPC (*t*(38) = 3.82, *P* = 0.02, peak: −24, −63, 42), but not in the HC or MPFC (*t*s(38) < 1.7, *P*s > 0.6). However, while the groups did not differ on PPC subsequent memory effects (*t*(38) < 1.9, *P* > 0.5), the control group exhibited a significantly stronger relationship between LPFC encoding activity and subsequent memory than the ASD group (*t*(38) = 3.82, *P* = 0.026, peak: −27, 51, 12), which was due to the fact that only the control group showed a significant subsequent memory effect in this region (*t*(19) = 3.91, *P* = 0.047, peak: −48, 36, 9), not the ASD group (*t*(19) = 2.1, *P* = 0.48). Therefore, the degree to which that activity in LPFC predicted retrieval success was reduced in the ASD group (see Fig. 3*A*).

During the memory retrieval task, both groups exhibited increases in activity relative to baseline in LPFC (*t*(38) = 8.43, *P* < 0.001, peak: −42, 36, 21) and PPC (*t*(38) = 12.94, *P* < 0.001, peak: −24, −63, 42), and marginally so in HC (*t*(38) = 3.11, *P* = 0.061, peak: −30, −27, −24), but not in MPFC (*t*(38) < 0.5, *P* > 0.8). However, even though LPFC activity was significantly increased in both the control (*t*(19) = 6.82, *P* < 0.001, peak: −48, 39, 15) and the ASD group (*t*(19) = 5.29, *P* = 0.003, peak: −42, 36, 21), BOLD activity in this region was higher in the control group compared with the ASD group during memory retrieval (*t*(38) = 3.34, *P* = 0.05, peak: −39, 33, 9). Activity during memory retrieval did not differ between groups in any other ROIs (*t*s(38) < 2.8, *P*s > 0.1). Therefore, lateral frontal activity was attenuated during the memory retrieval task, regardless of retrieval success or precision, in the ASD group relative to the control group (see Fig. 3*B*).

Within the memory retrieval task, further contrasts were conducted to investigate neural activity associated with retrieval success and retrieval precision. Increases in BOLD activity during successful relative to unsuccessful retrieval were found in HC (*t*(38) = 5.73, *P* < 0.001, peak: −30, −15, −12) and MPFC (*t*(38) = 4.08, *P* = 0.009, peak: −9, 45, −9), and neither of these effects differed between groups (*t*s(38) < 1.5, *P*s > 0.5). Retrieval success effects in PPC and LPFC did not reach significance (*t*s(38) < 3.0, *P*s > 0.08), and neither did group differences in these ROIs (*t*s(38) < 2.2, *P*s > 0.3). Activity in PPC showed a significant correlation with precision (*t*(38) = 3.67, *P* = 0.019, peak: −45, −63, 39), and this effect did not differ between groups (*t*s(38) < 2.0, *P*s > 0.4). Activity in none of the other ROIs showed a significant correlation with retrieval precision (*t*s(38) < 2.9, *P*s > 0.1) or differed between groups (*t*s(38) < 1.3, *P*s > 0.7). Therefore, we found no evidence that changes in neural activity associated with memory retrieval success and precision differ between groups, despite some evidence for lateral frontal dysfunction in the ASD group in terms of the relationship between encoding activity and subsequent memory as well as attenuated activity during memory retrieval. Exploratory whole-brain analyses (see Supplementary Material) also revealed minimal significant differences in BOLD activity associated with memory encoding and retrieval between the ASD and control groups in other regions of the brain.

### Functional Connectivity During Memory Encoding and Retrieval

Analyzing differences in BOLD activity can inform us about differences in regional function but does not provide information about how these regions communicate with other areas of the brain. It is possible that group differences in connectivity strength exist in the current data, even in areas for which no BOLD differences were found. To analyze functional connectivity during the memory encoding and retrieval tasks in the ASD and control groups, subject-specific seed regions (within the a priori defined ROIs) were created based on the location of peak activity during the memory retrieval task for LPFC, peak activity during successful (relative to unsuccessful) memory retrieval for MPFC and HC, and peak correlation between activity and retrieval precision for PPC, to best reflect the function of these regions in each participant. For each seed, the mean beta series was first correlated with 225 nodes across the whole brain to calculate each seed's level of whole-brain connectivity strength (node strength: see Methods) during the memory encoding and retrieval tasks.

**Figure 4.**
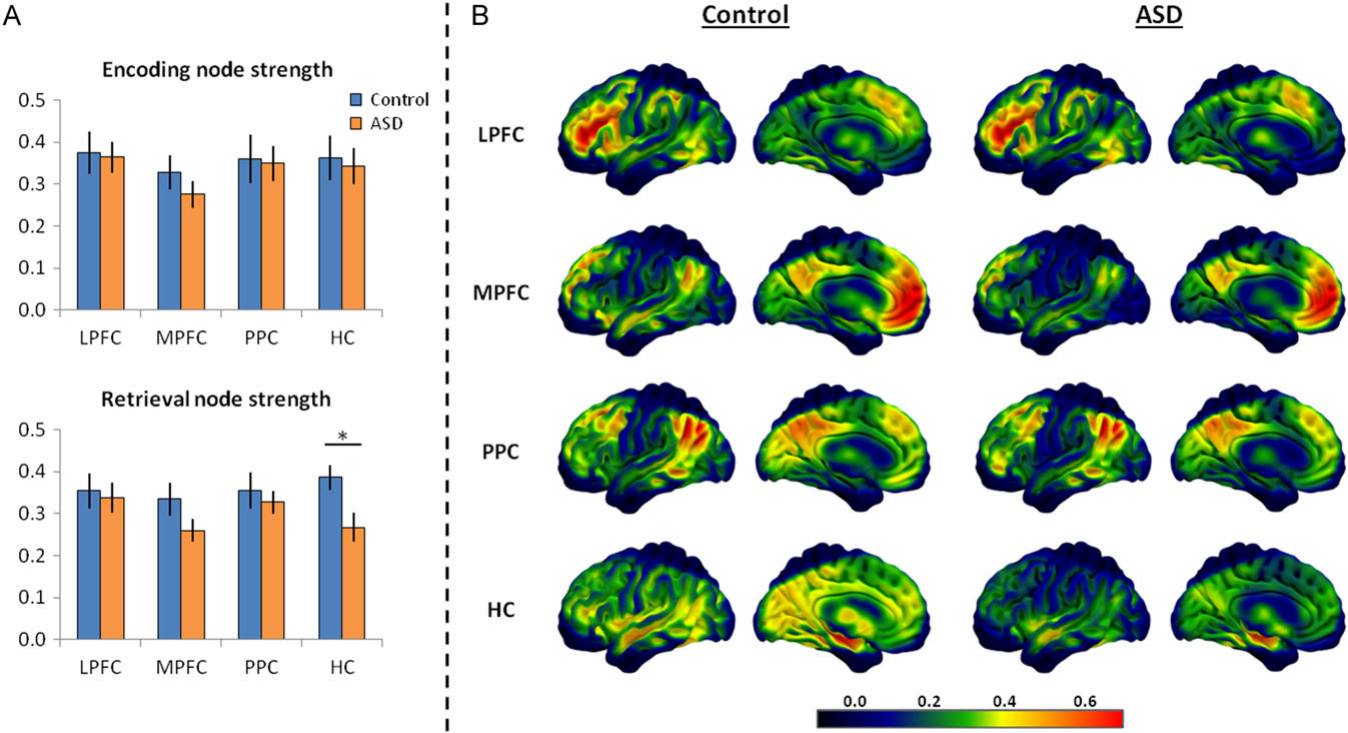
(*A*) The summary node strength measure for each of the 4 seed regions during the memory encoding and retrieval tasks in the control and ASD groups where the ASD group shows significantly reduced HC node strength, only during memory retrieval. (*B*) Smoothed heat maps illustrating each group's mean correlation between activity of each seed region during the memory retrieval task and regions across the whole brain (summarized as “node strength” for each seed). These images illustrate the general similarity between groups in whole-brain connectivity for the LPFC, MPFC, and PPC seeds, but a marked reduction in HC connectivity strength in the ASD group during memory retrieval. Effects are displayed using SurfIce.

When analyzing whole-brain connectivity during the memory encoding task, no evidence for between-group differences in overall communication strength between any of the seed regions and the rest of the brain were found (*t*s(38) < 1.1, *P*s > 0.31). During the memory retrieval task, no between-group differences in node strength were found for LPFC and PPC (*t*s(38) < 0.55, *P*s > 0.59) as well as for MPFC despite a numerical reduction (*t*(38) = 1.58, *P* = 0.12); however, the ASD group showed significantly reduced HC connectivity strength (*t*(38) = 2.63, *P* = 0.01) relative to the control group, thus suggesting reduced efficiency of HC communication within a whole-brain network. To ascertain if connectivity strength was significantly reduced during memory retrieval in the ASD group even when accounting for connectivity during the encoding task, node strength during encoding was included as a covariate when analyzing differences in node strength at retrieval. HC encoding node strength was a significant predictor of HC retrieval node strength (*F*_1,37_ = 10.33, *P* = 0.003), but, notably, HC connectivity was substantially reduced in the ASD group during retrieval even after accounting for connectivity during encoding (*F*_1,37_ = 7.74, *P* = 0.008). Therefore, memory retrieval was associated with a reduction in communication strength between the HC and the rest of the brain in the ASD group (see Fig. 4). This is particularly striking given the similarity in HC BOLD activity associated with successful retrieval between the 2 groups.

Measuring whole-brain node strength provides an indication of a particular seed region's general level of connectivity during a task, and has demonstrated that HC connectivity is reduced during memory retrieval in ASD, but this approach does not address the question of whether connectivity differences are driven by interactions with specific networks that are thought to be involved in memory retrieval. Specifically, it is possible that connectivity between the HC and either the FPCN and/or DMN may be predominantly disrupted in ASD. Therefore, to follow up on the group difference in memory retrieval-related HC connectivity strength, connectivity between the HC and the DMN and FPCN networks was analyzed. To maximize the fit of the networks assigned by Power et al. (2011) to our data and to ensure that the DMN and FPCN were distinct, the average within-network connectivity and between-network connectivity across all participants and tasks was calculated for every node. Nodes where within-network connectivity strength (*C*) was not at least 0.05 higher than between-network connectivity were removed from analyses for all participants. Note that the pattern of group differences did not change if other criteria were used.

**Figure 5.**
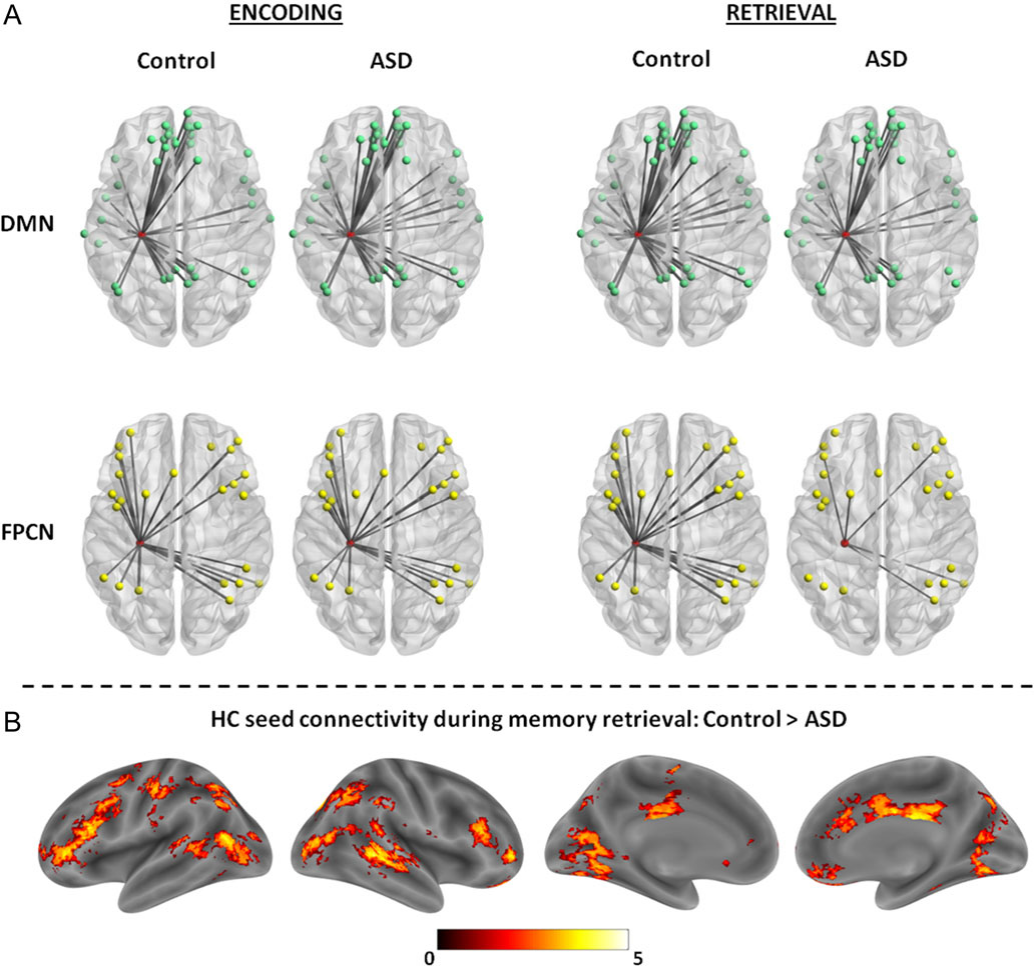
(*A*) Images depict connections (mean *r* > 0.25), with the width of the connection lines weighted by the mean correlation strength, between the HC (node in red) and the DMN (top) and FPCN (bottom) nodes during the memory encoding and retrieval tasks in both the ASD and control groups. The connections between the HC node and the network nodes illustrate the similarity in connectivity during memory encoding but a reduction in HC-network connectivity during memory retrieval in the ASD group, with substantial reductions in connectivity between the HC and FPCN. Images were generated using BrainNet Viewer. (*B*) Regions showing significantly higher HC connectivity in the control group compared with the ASD group during memory retrieval. Effects are displayed using bspmviewer at a threshold of *P* < 0.01 uncorrected for visualization, with the scale reflecting *t* values.

It was observed that the ASD group showed a substantial reduction in connectivity between HC and the FPCN (*t*(38) = 3.24, *P* = 0.002) and a marginal reduction in HC–DMN connectivity (*t*(38) = 1.88, *P* = 0.07) compared with the control group during the memory retrieval task, but, as expected based on the whole-brain node strength results, HC-network connectivity did not differ between groups during memory encoding (DMN: *t*(38) = 0.53, *P* = 0.60; FPCN: *t*(38) = 0.39, *P* = 0.70). Thus, despite the observation that HC connectivity reductions seem most pronounced with regions of the FPCN (see Fig. 5*A*), connectivity differences do not appear to be fully associated with a particular network. Therefore, to investigate the specific regions exhibiting attenuated HC connectivity during memory retrieval in ASD, a seed-to-voxel whole-brain connectivity analysis was conducted between the HC seed and the rest of the brain, using a threshold of *P* < 0.001, minimum extent of 20 voxels, to identify regional differences in connectivity between the 2 groups (see Table 3). Interestingly, the regions showing significantly reduced connectivity with HC in the ASD group include those typically associated with the DMN (e.g. middle temporal gyrus) and those associated with the FPCN (inferior/middle frontal gyrus), as well as the caudate and middle cingulate gyrus (see Fig. 5*B*). In contrast, no significant differences were observed for HC seed-to-voxel connectivity between groups during the memory encoding task. There were also no regions for which HC connectivity was significantly greater in the ASD group compared with the control group during both memory encoding and retrieval. Thus, reductions in HC functional connectivity seem to be relatively widespread in ASD during memory retrieval, but are perhaps most pronounced with regions within the FPCN such as inferior/mid frontal cortex.

**Table 3:** Regions exhibiting reduced connectivity with the HC seed during memory retrieval in the ASD group relative to the control group

Region	Peak *t*	*x*	*y*	*z*	*k*
R mid temporal gyrus	5.14	48	−42	3	55
R orbital superior frontal gyrus	5.01	15	54	−15	42
L inferior/mid frontal gyrus	4.47	−36	24	18	43
R mid cingulate gyrus	4.17	6	−21	27	36
L caudate	3.99	−9	9	9	29

A series of control analyses were conducted to test whether the finding of reduced retrieval-related HC connectivity in the ASD group may have been influenced by analysis or task factors, where it was confirmed that a retrieval-related HC connectivity reduction was not affected by the choice of contrast to select seed regions, the number of trials analyzed, and the choice of threshold to calculate node strength (see Supplementary Material).

## Discussion

In the present study, we used fMRI to investigate the neural basis of putative differences in episodic memory encoding and retrieval in ASD, focusing on activity and functional connectivity of episodic memory networks. We used a novel approach involving continuous measures of retrieval that allowed us to dissociate 2 components of recollection: retrieval success (the probability of successful recollection) and retrieval precision (the fidelity of successfully retrieved memories). Behaviorally, participants with ASD exhibited a reduction in instances of recollection success but we found no evidence for an additional reduction in the precision of successfully retrieved memories. Neurally, we observed comparable levels of activity and functional connectivity during the memory encoding task between the groups, but LPFC activity during encoding predicted subsequent memory only in the control group and not in the ASD group. Furthermore, while both groups showed similar patterns of activity in HC and MPFC associated with retrieval success and in PPC associated with retrieval precision, the ASD group exhibited reduced LPFC activity during the memory retrieval task. Notable differences were also found between the groups in functional connectivity during the memory retrieval task, in which the ASD group showed reduced HC node strength within a whole-brain network compared with the control group, and pronounced reductions in connectivity particularly between HC and FPCN regions, such as inferior/mid frontal cortex. Taken together, these findings suggest that episodic memory retrieval deficits in ASD arise from mechanisms that predominantly affect retrieval success via attenuated retrieval-related functional connectivity.

The reduction in recollection success in ASD observed here is consistent with the findings of previous studies that have used binary measures to assess instances of recollection in ASD, such as source memory (e.g. Bowler et al. 2004; Cooper et al. 2016) and remember versus know responses (e.g. Bowler et al. 2007; Cooper et al. 2015; Gaigg et al. 2015), demonstrating that recollection failures occur more frequently in ASD. Furthermore, the integrity of retrieval precision observed in the present data is consistent with previous studies demonstrating that recollection specificity can be manipulated similarly in ASD and typical controls (Bowler et al. 2007; Crane et al. 2012), suggesting that once a memory is recollected successfully it is experienced by individuals with ASD in a comparable way to typical individuals. Notably, we found that posterior parietal activity correlated with retrieval precision, with no evidence for differences in activity or functional connectivity of this region during memory retrieval between the ASD and control groups. These findings are at odds with the theory that parietal dysfunction might underpin recollection deficits in ASD (Boucher and Mayes 2012) and suggest that the representation of memory details, thought to involve posterior parietal cortex (e.g. Kuhl and Chun 2014; Bonnici et al. 2016), may differ little in individuals with ASD and typical individuals. However, it is important to note that we did not find evidence for a “disproportionate” reduction in retrieval success, and therefore any conclusions about “intact” retrieval precision and lateral parietal function in ASD are tentative and must be further investigated in different task contexts. Moreover, a limitation is that our design did not enable us to assess representational specificity of individual memories within PPC, which would provide a further valuable piece of evidence to assess the precision of successfully recollected memories in ASD.

Some previous explanations of memory deficits in ASD focus on the manner in which information is encoded (e.g. Bowler et al. 2014; Meyer et al. 2014); however, we observed no overall enhancement or attenuation of activity in any region in the ASD group compared with the control group while participants were encoding the scenes (vs. baseline). In particular, we found no evidence for between-group differences in overall activity or functional connectivity of LPFC, previously linked with selection and organization of information during encoding (Blumenfeld and Ranganath 2007), and HC, thought to be involved in relational encoding (Konkel and Cohen 2009). Notably, however, activity in LPFC predicted subsequent retrieval success only in the control group, not in the ASD group, a finding that was also apparent in inferior temporal cortex following a whole-brain analysis. This finding partially supports that of previous fMRI research (Gaigg et al. 2015) as well as our recent observation of a dissociation between encoding-related eye movements and retrieval success in ASD (Cooper et al. 2017). Based on the lack of difference in overall LPFC activity during encoding, a reduced relationship between encoding activity (presumably a marker of encoding processes) and memory could suggest that operations at encoding are less likely to determine retrieval success in ASD compared with typical controls, implying that retrieval processes may have a more pronounced influence on the episodic memory deficits observed here. It is important to emphasize, however, that both enhanced and attenuated lateral frontal activity have been previously observed in ASD during memory encoding (Greimel et al. 2012; Gaigg et al. 2015). Thus, it is important not to disregard potential differences in encoding in ASD and of course encoding and retrieval are largely interdependent processes.

In contrast to the results from the memory encoding phase, the ASD group showed reduced LPFC activity during the memory retrieval task (vs. baseline) compared with the control group, in line with other tasks that have shown a similar reduction (e.g. Koshino et al. 2008; Damarla et al. 2010; Solomon et al. 2015). The processes reflected in such frontal dysfunction during memory retrieval may relate to preretrieval search strategies or postretrieval monitoring, resolving conflict between memory representations, both of which have been associated with LPFC (Badre and Wagner 2007). There is some evidence for a deficit in postretrieval monitoring in ASD such that, in free recall tasks, individuals with ASD not only recall fewer correct details but can also generate more incorrect details (Maras and Bowler 2011; Maras et al. 2013). A postretrieval monitoring difficulty would also explain why providing more specific retrieval cues aids performance in subjects with ASD (Bowler et al. 2004; Maras et al. 2013) and account for findings of reduced metamemory, monitoring the accuracy of memory decisions, as has been previously reported in ASD (e.g. Grainger et al. 2014; Cooper et al. 2016). A deficit in controlled retrieval processes could explain why we did not find LPFC retrieval activity to vary according to retrieval success in either group. According to Badre and Wagner (2007), left midventrolateral prefrontal cortex subserves a domain general process of controlled retrieval that is required with increasing specificity and demands of retrieval goals (Velanova et al. 2003), such as during recollection and source memory attempts (Dobbins and Wagner 2005; Gallo et al. 2010) over and above item recognition. Therefore, LPFC activity may be particularly reflective of a recollection attempt, perhaps suggesting that individuals with ASD were less able to initiate a recollection-based strategy to improve retrieval success.

A number of studies have speculated on the brain regions that might be implicated in the pattern of memory deficits observed in ASD, mostly focusing on prefrontal and hippocampal dysfunction as possible candidates (e.g. Bowler et al. 2010; Solomon et al. 2011). Notably, the control and the ASD groups exhibited similar increases in hippocampal activity associated with successful memory retrieval, replicating a robust finding in the literature within the typical population (cf. Eichenbaum et al. 2007; Spaniol et al. 2009; Vilberg and Rugg 2012), and thought to reflect the involvement of the HC in the reactivation of memory representations (Simons and Spiers 2003). In the current study, we thus observed direct evidence of reduced frontal but not hippocampal activity during episodic memory retrieval in ASD, supporting recent fMRI evidence for such a pattern in a relational learning task (Solomon et al. 2015) as well as behavioral evidence pointing to frontal dysfunction rather than hippocampal dysfunction during episodic memory retrieval in ASD (Solomon et al. 2011, 2016; Cooper et al. 2015). However, while these findings provide important elements to improve our understanding of neural differences between individuals with ASD and typical adults, it is beneficial to move away from a strict modular approach focusing on frontal dysfunction in ASD, for example, to one that aims to understand network dynamics (cf. Barendse et al. 2013) across the episodic memory network.

Interestingly, while HC activity did not differ between the 2 groups, the ASD group showed a substantial decrease in HC node strength, reflective of whole-brain connectivity. Interestingly, this group difference in HC functional interactivity was specific to the memory retrieval task and was not present during memory encoding, which could mirror the difficulty individuals with ASD experience in engaging in the retrieval process of recollection. The current findings add to the body of evidence which suggests that reduced functional connectivity is related to many differences in information processing in ASD (Just et al. 2012), such as during working memory tasks (Koshino et al. 2008; Solomon et al. 2009; Damarla et al. 2010). The findings also tie in with what is known about brain region interactions during memory retrieval in the typical population. Both the prefrontal cortex and HC are hubs of high connectivity during recollection and the HC in particular is thought to increase its connections to regions across the whole brain dynamically, reflecting increased transfer and integration of information during memory retrieval (Schedlbauer et al. 2014; Geib et al. 2015). Moreover, the strength of whole-brain hippocampal connectivity, reflecting the efficiency of communication with regions across the brain, is associated with recollection-based memory performance (Schedlbauer et al. 2014; Geib et al. 2015; King et al. 2015). This evidence highlights the importance of HC whole-brain connectivity integrity to episodic memory retrieval and emphasizes the potential implications for recollection in ASD if the medial temporal lobe is not acting as a hub within a whole-brain network during memory retrieval in this population.

Even though the HC showed widespread reduction in connectivity during memory retrieval in ASD, some of the most substantial reductions appeared to be with FPCN regions, such as inferior/middle frontal gyrus. Communication between the HC and regions of the FPCN is thought to underpin the monitoring of recollected information and is increased in instances of “recall-to-reject” responses (Bowman and Dennis 2016), emphasizing the importance of these connections in governing top-down control of recollection-based retrieval. Similarly, functional connectivity between the HC and inferior frontal gyrus is stronger during successful memory retrieval (Hannula and Ranganath 2009) and has been shown to increase as a function of mnemonic load during working memory (Rissman et al. 2008), and as a function of cognitive control demands during source memory retrieval (Barredo et al. 2013). Interaction between these 2 regions also increases as a function of memory vividness for external perceptual details (Ford and Kensinger 2016). Therefore, hippocampal connectivity with the FPCN seems to be important for facilitating successful and detailed explicit recollection (cf. Simons and Spiers 2003; Fornito et al. 2012), which appears to be disrupted in ASD. Of note, however, regions showing reduced hippocampal connectivity in ASD were not restricted to the FPCN, but also included middle temporal gyrus, middle cingulate cortex, and the caudate, regions that have previously been associated with cognitive and behavioral flexibility difficulties in ASD (Solomon et al. 2009; D'Cruz et al. 2016). All 3 of these regions commonly show recollection-related increases in activity in neurotypical individuals (Spaniol et al. 2009; Kim 2016) and, alongside inferior frontal gyrus, exhibit some of the strongest increases in HC connectivity during vivid memory retrieval (Geib et al. 2015). It is notable therefore that the regions exhibiting attenuated HC connectivity in ASD are those for which hippocampal connectivity is strongest and most important for successful recollection in neurotypical individuals.

The current findings perhaps suggest that memory representations are processed and activated by the HC in a similar way in ASD and controls during successful retrieval, but are not searched for, transferred, or monitored in an efficient way during episodic memory retrieval as a result of widespread disrupted connectivity. However, caution must be applied when inferring cognitive processes from connectivity and activity differences and further studies are needed to clarify the specific processes that may be disrupted during episodic memory retrieval in ASD. Moreover, it is emphasized that a focus on frontal dysfunction or even individual region-to-region connections is highly unlikely to fully explain the basis of memory deficits in this population. However, given the poor temporal resolution of fMRI, a limitation with the current study is that we cannot conclude at which stage reduced connectivity is most pronounced and whether preretrieval or postretrieval processes may be more responsible for memory deficits. Investigating the neural processes of episodic memory retrieval in ASD via electroencephalography/magnetoencephalography (EEG/MEG) may provide additional insight to address this important question (cf. Bergström et al. 2013). Only 2 such studies have been conducted in ASD, reporting evidence for attenuated frontal old–new effects during both early and late stages of recognition memory trials (Massand et al. 2013) and time nonspecific old–new effects in ASD compared with controls (Massand and Bowler 2013). Differences in neural connectivity using EEG/MEG have not been studied to date in ASD during memory retrieval and would provide a valuable future study to explore the role of functional underconnectivity in episodic memory deficits in ASD.

In conclusion, by combining a novel memory-precision paradigm with fMRI, our study provides important insights into the behavioral and neural characteristics of memory deficits associated with ASD. Our data demonstrate that reduced overall retrieval success in ASD is accompanied by diminished connectivity during memory retrieval, particularly between the HC and whole-brain networks such as the FPCN. These results strengthen the view that memory deficits in the ASD are driven by retrieval-related impairments that reduce the probability of recollection success, and highlight the importance of investigating network connectivity in contrast to more modular approaches in helping to understand memory deficits in ASD.

## Supplementary Material

Supplementary material are available at *Cerebral Cortex* online.

## Funding

This work was supported by a James S. McDonnell Foundation Scholar Award to J.S.S. and was carried out within the University of Cambridge Behavioural and Clinical Neuroscience Institute, funded by a joint award from the Medical Research Council and the Wellcome Trust. R.A.C. was funded by the Economic and Social Research Council and P.M.B. by the Wellcome Trust.

## Supplementary Material

Supplementary Data
