# Supplementary material for: Reduced Hippocampal Functional Connectivity During Episodic Memory Retrieval in Autism
Source: Cereb Cortex. 2017 Jan 5;27(2):888–902. doi: 10.1093/cercor/bhw417 (PMC5390398; doi:10.1093/cercor/bhw417)
Supplement: Supplementary Data [file Supplemental_R2.docx]

**Supplementary materials**

**Supplementary Results**

***BOLD activity: whole-brain contrasts***

To follow-up on the ROI analyses of memory- and group-related differences in BOLD activity, we also checked for any other significant changes in activity across the whole brain (see Table S1). Main effects of encoding and retrieval minus baseline were analysed at a strict threshold of *p* < .01 FWE corrected, minimum extent of 20 voxels, due to the expected extent of change in activity. All other contrasts were analysed at a more lenient threshold of *p* < .001 uncorrected, minimum extent of 20 voxels. Memory encoding and retrieval (versus baseline) were largely associated with regions typically associated with top-down attention and visual perception, such as occipital and parietal cortices and lateral frontal cortex, with memory retrieval additionally recruiting regions such as the insula and putamen. Minimal differences in BOLD activity were identified between the groups. Activity during encoding that positively predicted subsequent memory success was similarly observed in regions of occipital, inferior temporal, and lateral frontal cortices, with inferior temporal activity appearing to be a stronger predictor of subsequent memory success in the control group compared to the ASD group. Activity associated with successful memory retrieval was identified in a network of medial temporal and sub-cortical regions, such as the hippocampus and caudate, as well as regions such as medial frontal cortex and mid temporal cortex, typically associated with the default mode network (DMN). In contrast, memory precision was associated with bilateral activity in inferior temporal and inferior parietal cortices. No group differences were observed for activity associated with memory success and memory precision.

*Table S1. Significant results from whole-brain contrasts, including main effects of each memory contrast and differences between groups.*

| Contrast | Region | Peak t | x | y | z | k |
| --- | --- | --- | --- | --- | --- | --- |
| Encoding - Baseline | Bilateral occipital cortex, inferior and medial temporal lobe | 21.19 | 42 | -72 | -12 | 6088 |
|  | L inferior frontal gyrus precentral gyrus, mid frontal gyrus | 9.56 | -45 | 3 | 33 | 912 |
|  | Bilateral supplementary motor area, superior frontal gyrus | 8.83 | -6 | 9 | 57 | 97 |
|  | R inferior frontal gyrus, mid frontal gyrus | 7.94 | 54 | 33 | 27 | 63 |
|  | R inferior frontal gyrus, precentral gyrus | 7.49 | 51 | 9 | 30 | 72 |
|  | R mid frontal gyrus | 7.27 | 30 | 0 | 51 | 33 |
| *Control > ASD* | R supramarginal, precentral gyrus | 4.20 | 60 | -21 | 42 | 40 |
| *ASD > Control* | - | - | - | - | - | - |
| Subsequent memory | R occipital and inferior temporal cortex | 6.45 | 45 | -72 | -12 | 1246 |
|  | L occipital and inferior temporal cortex | 6.17 | -48 | -69 | -9 | 1010 |
|  | Bilateral supplementary motor area, superior frontal gyrus | 5.33 | -6 | 6 | 60 | 137 |
|  | L middle frontal gyrus, precentral gyrus | 5.19 | -51 | 3 | 48 | 136 |
|  | L inferior frontal gyrus | 4.70 | -51 | 33 | 6 | 49 |
|  | L superior frontal gyrus | 4.44 | -24 | -3 | 60 | 75 |
| *Control > ASD* | L inferior temporal cortex | 4.47 | -57 | -39 | -18 | 42 |
| *ASD > Control* | - | - | - | - | - | - |
| Retrieval - Baseline | Bilateral occipital, inferior temporal, inferior parietal cortices | 21.99 | 33 | -87 | 12 | 7086 |
|  | L inferior frontal gyrus, mid frontal gyrus, precentral gyrus | 12.79 | -51 | 6 | 33 | 908 |
|  | R mid frontal gyrus, precentral gyrus, inferior frontal gyrus | 11.06 | 54 | 12 | 33 | 309 |
|  | R insula and putamen | 10.39 | 30 | 18 | 6 | 276 |
|  | R inferior frontal gyrus, mid frontal gyrus | 9.80 | 48 | 30 | 24 | 190 |
| *Control > ASD* | Bilateral cuneus | 3.80 | 9 | -78 | 24 | 45 |
| *ASD > Control* | - | - | - | - | - | - |
| Successful > Unsuccessful Retrieval | Bilateral caudate, insula, postcentral gyrus, hippocampus, parahippocampal gyrus, medial frontal cortex | 7.16 | -6 | 6 | -6 | 2697 |
|  | R precentral gyrus | 5.37 | 60 | 9 | 6 | 53 |
|  | R supramarginal | 4.93 | 63 | -18 | 21 | 212 |
|  | L mid temporal cortex | 4.86 | -66 | -24 | -15 | 79 |
|  | L precentral gyrus | 4.26 | -24 | -24 | 57 | 47 |
|  | Bilateral supplementary motor area | 3.91 | 6 | -6 | 51 | 94 |
|  | L cuneus | 3.90 | -9 | -81 | 24 | 23 |
| *Control > ASD* | - | - | - | - | - | - |
| *ASD > Control* | - | - | - | - | - | - |
| Precision | R inferior temporal cortex | 5.56 | 54 | -24 | -21 | 73 |
|  | R inferior parietal cortex | 5.15 | 51 | -51 | 51 | 23 |
|  | L inferior and mid temporal cortex | 5.14 | -66 | -30 | -15 | 292 |
|  | L inferior parietal cortex and angular gyrus | 4.49 | -57 | -48 | 48 | 94 |
| *Control > ASD* | - | - | - | - | - | - |
| *ASD > Control* | - | - | - | - | - | - |

***Functional connectivity additional analyses***

*Control analyses*

We conducted a series of control analyses to verify that the retrieval-related reduction in HC connectivity in the ASD group was not driven by analysis or task factors (see Table S2 for a summary of results). The influence of each factor on HC node strength was assessed for both the memory encoding and retrieval tasks. First, the threshold for defining ‘connections’ was varied from the threshold of r > .25 used for the main analysis to measure HC node strength based on a connection threshold of r > .1 and r > .5 during both encoding and retrieval. A higher threshold resulted in low values of node strength (as more connections are assigned a value of 0), but the pattern of selectively reduced connectivity during retrieval in the ASD group did not change. The next analysis controlled for the difference in trial numbers between the encoding and retrieval tasks, where a greater number of retrieval trials could potentially result in a more sensitive analysis for detecting differences in connectivity. Node strength was thus calculated based on selecting a random subset of retrieval trials to match the number of encoding trials. Doing so also did not change the selective reduction in HC retrieval connectivity. The final control analysis addressed the location of seed regions and specifically how seeds were selected based on peak retrieval-related effects. Thus, activity within seed regions may not be sensitive to encoding processes, leading to an apparent selective reduction in connectivity during retrieval. Analyses were repeated using HC seed regions based on participants’ peak encoding-related HC activity, and again revealed evidence for reduction in HC node strength during memory retrieval but not during memory encoding.

*Table S2. Mean HC node strength during encoding and retrieval in the ASD and control groups for the four control analyses.*

| Control analysis | Encoding HC connectivity | | | Retrieval HC connectivity | | |
| --- | --- | --- | --- | --- | --- | --- |
|  | Control | ASD | *p* | Control | ASD | *p* |
| Threshold = r > .1 | 0.39 (0.23) | 0.37 (0.18) | .75 | 0.41 (0.11) | 0.31 (0.14) | .01 |
| Threshold = r > .5 | 0.23 (0.25) | 0.22 (0.20) | .87 | 0.18 (0.12) | 0.10 (0.11) | .03 |
| Matched trial numbers | 0.36 (0.24) | 0.34 (0.19) | .77 | 0.38 (0.16) | 0.28 (0.14) | .04 |
| Encoding-specific seeds | 0.35 (0.25) | 0.31 (0.16) | .50 | 0.41 (0.16) | 0.26 (0.14) | .004 |

*Connectivity within and between the DMN and FPCN*

In addition to analysing seed-to-network connectivity, we also analysed within-network connectivity (network density) and between-network connectivity (degree to which the networks communicate or are modular) to analyse the function of these networks in ASD beyond specific relationships with our ROIs. Within-network connectivity strength was defined as the sum of the strength of all within-network ‘connections’ divided by the total possible number of within-network connections. Similarly, between-network connectivity strength was defined as the sum of the strength of all between-network ‘connections’ divided by the total possible number of between-network connections. During the memory encoding task, no between-group differences were found in network density for both DMN (*t*(38) = 0.26, *p* = .80) and FPCN (*t*(38) = 0.07, *p* = .95). Differences in within-network density also did not reach significance during the memory retrieval task for both DMN (*t*(38) = 1.33, *p* = .19) and FPCN (*t*(38) = 0.69, *p* = .49). Similarly, the group differences in between-network connectivity did not reach significance during memory encoding (*t*(38) = 1.29, *p* = .20) or memory retrieval (*t*(38) = 1.88, *p* = .07) despite a numerical reduction in between network connectivity in ASD (also seen visually in Figure S1).


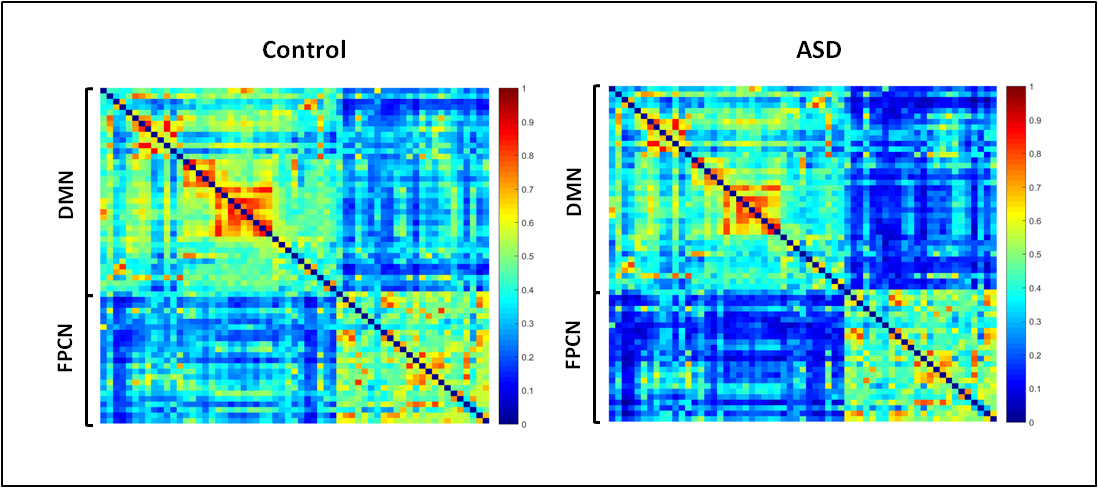


*Fig. S1. Correlation matrices depicting the within network (top left and bottom right squares) and between network (bottom left and top right squares) mean node correlations for the DMN and FPCN in the control and ASD groups during the memory retrieval task.*
